# Targeted delivery of doxorubicin by nano-loaded mesenchymal stem cells for lung melanoma metastases therapy

**DOI:** 10.1038/srep44758

**Published:** 2017-03-17

**Authors:** Yuekui Zhao, Shanshan Tang, Jiamin Guo, Murad Alahdal, Shunxiu Cao, Zhaocong Yang, Fangfang Zhang, Yumeng Shen, Minjie Sun, Ran Mo, Li Zong, Liang Jin

**Affiliations:** 1 State Key Laboratory of Natural Medicines, School of Life Science and Technology, China Pharmaceutical University, Nanjing 210009, China; 2 State Key Laboratory of Natural Medicines, Department of Pharmaceutics, China Pharmaceutical University, Nanjing 210009, China

## Abstract

Poor antigenic presentation of tumor tissues and a lack of specific targets currently limit the success of nanoparticle delivery system. Cellular carrier technique has been recently explored extensively as a substitutive or supplement for traditional targeting delivery system. Here, we demonstrate the usage of mesenchymal stem cells (MSCs) loaded with doxorubicin containing polymer nanoparticles in pulmonary melanoma metastases therapy, as a modified technique of targeted delivery system. The characterizations of prepared nanoparticles and MSCs sensitivity to DOX and PLGA-DOX were measured. *In vitro* tumor tropism, and *in vivo* distributions of nanoparticles loaded MSCs were also investigated. The findings have demonstrated that, the modified system not only integrates the controlled-release property of nanoparticles but also exhibits tumor tropism and penetrative characteristics of MSCs. Furthermore, the *in vitro* and *in vivo* anti-tumor study has demonstrated that drug loaded MSCs had potent efficacy in lung melanoma metastases treatment.

Despite medical technology progress and novel drug discoveries, the incidence of cancer mortality is still on the rise^1^. As the leading cause of malignancy-associated death, metastatic cancers, especially lung metastasis, are almost incurable owing to their systemic feature, drug resistance and low targeting efficiency^2^,^3^. Conventional chemotherapy and radio-therapeutic approaches remains the mainstay for treating primary or metastasizing tumor cells. Nanocarriers, such as liposome^4^ and micelle^5^ have also demonstrated some limited success in treating certain cancer types depending on the enhanced permeation and retention (EPR) effect. However, the outcome of metastatic lung cancer therapy remains poor due to limited targeting efficiency of lung tumor cells and a lack of target selectivity between tumor cells and normal cells^6^. Therefore, lung metastasis remains one of the refractory diseases that demands innovative drug delivery system urgently.

Recently, cell-based therapies have attracted a great deal of attention as promising alternatives for cancer treatments^7^. Mesenchymal stem cells (MSCs) in particular are viewed as having the greatest therapeutic potential. MSCs can be isolated from number of sources, initially from bone marrow, and more recently from adipose tissue, and umbilical cord^8^,^9^. The bone marrow extraction of MSCs produces low yields and the procedure itself was found painful and invasive. Hence, adipose tissue-derived MSCs have become popular in the medical research due to the minimally invasive process and high yield^10^. MSCs used in our research were isolated from adipose tissue of C57BL6 mice. It is worthy mentioned that MSCs not only possess low immunogenicity^11^ but they also can home to disseminated metastatic lesions and track down malignant cells^12^,^13^,^14^. Therefore, MSCs are considered as “magic bullets” for targeted delivery of genes or chemotherapeutic agents.

In the past few years, engineered MSCs have been widely studied for the delivery of therapeutic cytokines including interleukin-2 (IL-2)^15^, IL-12^16^, interferon-β (IFN-β)^17^, all of which exhibited efficient anti-tumor effects. Nevertheless, the safety problems in transduction with viral vectors and efficiency issues in non-viral vectors remain in consideration^18^. Hence, MSCs engineered with chemotherapeutics have been widely established for their tumor tropic delivery. However, MSCs overexpression of drug efflux pumps often hampers intracellular accumulation of small molecule therapeutics^19^.

In this paper, we supposed that, doxorubicin (DOX) containing nanoparticles that were formulated with poly (d, l-lactic-co-glycolic acid) (PLGA) will achieve successful drug retention in MSCs. MSCs derived from C57BL6 mice adipose tissue and loaded with PLGA-DOX nanoparticles (NP-MSCs) could target lung metastases in C57BL6 mice, suggesting a more effective methodology to treat lung metastases tumor (Fig. 1).

## Results

### Physicochemical properties of PLGA-DOX nanoparticles

Particle size, polydispersity index (PDI) and zeta potential of PLGA-DOX and PLGA-DiR are presented in Table 1. PLGA-DOX nanoparticles were formulated using carboxymethyl cellulose as the surfactant. Carboxymethyl cellulose can also increase the number of carboxyl groups on nanoparticles making it possible to combine PLGA and DOX more tightly. As a result, DOX was efficiently encapsulated in nanoparticles. The encapsulation efficiency (EE) and drug loading coefficient (DL) was measured 90.52 ± 0.63% and 10.77 ± 0.91%, respectively.

**Figure 2:**
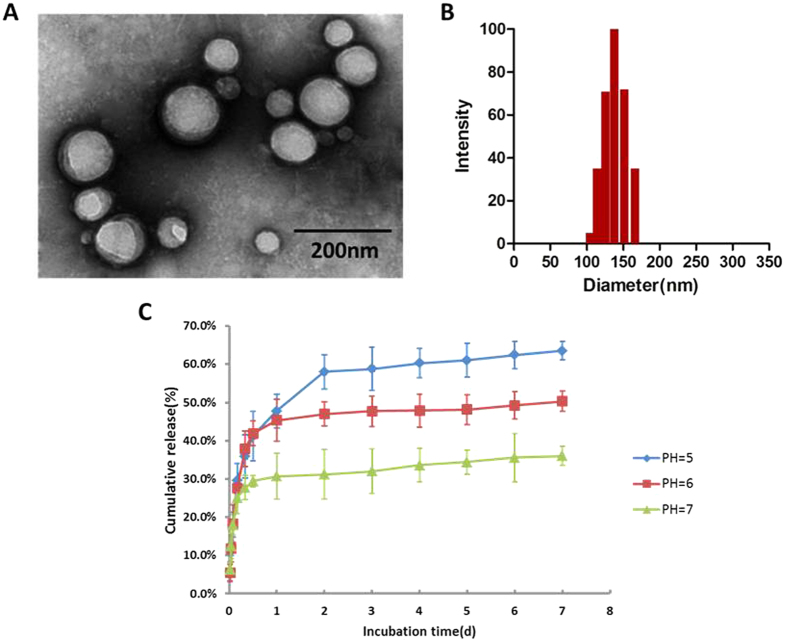
Characteristics of PLGA-DOX. (**A**) TEM image of PLGA-DOX nanoparticles. (**B**) PLGA-DOX nanoparticle size distribution. (**C**) *In vitro* release profile of DOX from nanoparticles at pH 5.0, 6.0 and 7.0, displaying the pH-sensitive DOX release. Data were presented as mean ± standard deviation of three independent experiments.

Transmission electron microscopy (TEM) image and the size distribution of PLGA-DOX were given in Fig. 2A,B, respectively. As the figures indicated, the nanoparticles have spherical morphology with a uniform size. *In vitro* release of DOX from nanoparticles is shown in Fig. 2C, illustrating an initial burst release in the first 4 hrs followed by a stable release phase.

### MSCs sensitivity to DOX and PLGA-DOX

**Figure 3:**
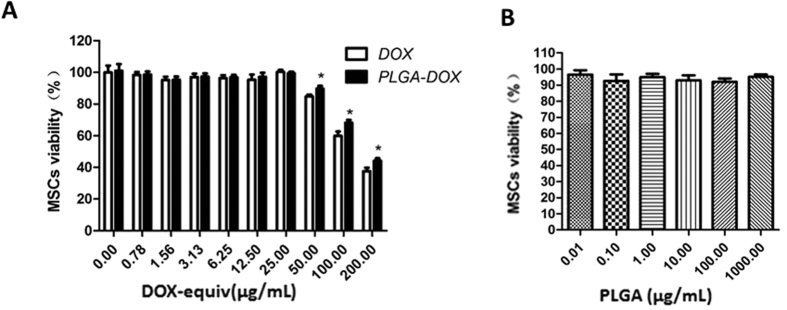
Influence of PLGA, DOX and PLGA-DOX on MSCs viability. MSCs were incubated with (**A**) free DOX and PLGA-DOX, (**B**) PLGA for 24 h at 37 °C to evaluate the MSCs sensitivity. Data were presented as mean ± standard deviation (n = 6 replicates for each group). *p < 0.05, compared to DOX group at the same concentration.

MSCs sensitivity to DOX, PLGA-DOX and PLGA were quantified. Drug-free PLGA nanoparticles were found to be rather benign toward the MSCs even at a high concentration of 1000 μg/mL by MTT assay (Fig. 3B). As shown in Fig. 3A, MSCs survival at 24 h was also unaffected after incubation with different concentration levels of DOX and PLGA-DOX ranging from 0.78 to 25.00 μg/mL (DOX-equiv). The MSCs viability in PLGA-DOX group remained 90.38 ± 2.52% even at a high concentration of 50 μg/mL, suggesting that PLGA-DOX was non-toxic to MSCs in certain extent. Nevertheless, a rapid decline of cell viability was found under the concentration of 200 μg/mL.

### MSCs uptake of PLGA-DOX

**Figure 4:**
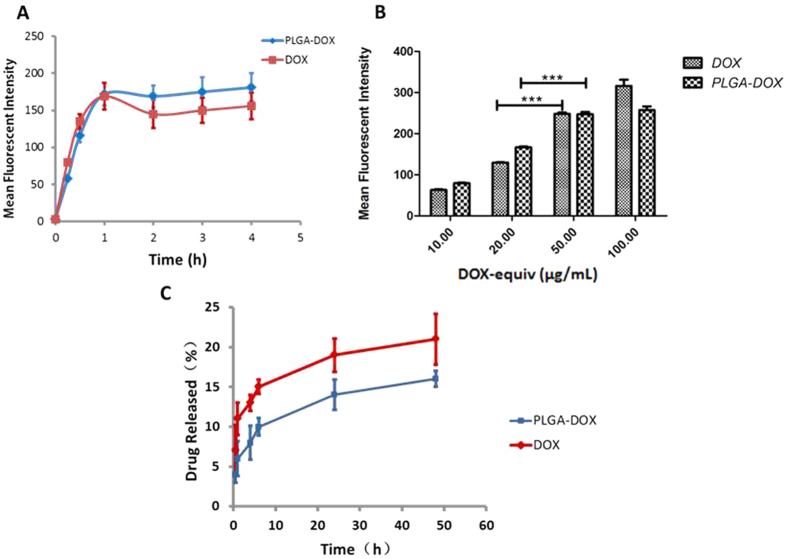
Cellular uptake of DOX and PLGA-DOX by MSCs and release profile of drug loaded MSCs. (**A**) displays the time dependent manner of MSCs uptake, and (**B**) reports the dose-dependent uptake of MSCs in 1 hr detected by flow cytometry. (**C**) Profile of drug released from NP-MSCs and DOX-MSCs. Data were presented as mean ± standard deviation of three independent experiments, *p < 0.05, **p < 0.01, ***p < 0.001.

To explore the optimal cellular uptake condition, qualitative assay for the effect of incubation time and concentration was conducted using flow cytometry. As shown in Fig. 4A, at the same concentration of 20 μg/mL (DOX-equiv), intracellular uptake was found to be time-dependent. The uptake of DOX and PLGA-DOX displayed a rapid intake in the first 1 hr, followed by negligible change subsequently, suggesting the saturation of incorporation (Fig. 4A). In another experiment, DOX uptake gradually increased in a concentration dependent manner under 1 hr incubation time (Fig. 4B). A significant increase in uptake was detected in PLGA-DOX group moving from 20 μg/mL to 50 μg/mL (DOX-equiv). However, at 100 μg/mL, PLGA-DOX incorporation displayed no obvious increase compared to 50 μg/mL.

Taking concentration and MSCs viability into account then, the most favorable condition would be co-incubating MSCs with nanoparticles for 1 hr at the concentration of 50 μg/mL (DOX-equiv).

The drug content loaded in MSCs was determined by HPLC. After 1 hr incubation with 50 μg/mL DOX or PLGA-DOX, 5 × 10^5^ drug-loaded MSCs was extracted for analysis. The average DOX content was measured 24.67 ± 5.33 pg/cell and 20.98 ± 4.02 pg/cell for DOX and PLGA-DOX group, respectively.

MSCs treated with DOX and PLGA-DOX (50 μg/mL, DOX-equiv) for 1 hr were used for drug release analysis. Drug released from NP-MSCs during the first 30 min was only 4.01 ± 1.05% followed by sustained release with approximately 16.28 ± 1.26% of drug release in 48 h. DOX released from MSCs in the first 30 min was significantly higher than in NP-MSCs group (*p* < 0.05).

### *In vitro* tumor tropism of MSCs

**Figure 5:**
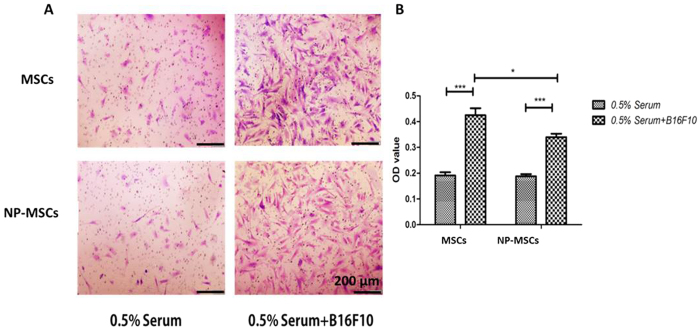
*In vitro* tumor tropism of MSCs. (**A**) Representative photographs of unloaded and loaded MSCs migration through the membrane pores. (**B**) OD_570_ value of stain crystal violet in each group. 0.5% Serum was used as control group. Data were presented as mean ± standard deviation, (n = 6 replicates for each group). *p < 0.05, **p < 0.01, ***p < 0.001.

To track the *in vitro* migration of MSCs towards B16F10 melanoma cells, a 24-well Transwell chamber was used. The number of migration MSCs significantly increased when compared with the 0.5% Serum control group (*p* < 0.001). As demonstrated in Fig. 5, the unloaded and loaded MSCs responsed to the B16F10 in the bottom chamber and migrated through the membrane pore, demonstrating their tumor tropism toward tumor cells. Although the migration behavior of loaded MSCs toward B16F10 showed statistical reduction in cell number (*p* < 0.05), their B16F10 tropism trait was noted to be similar to unloaded MSCs and also adequate for drug delivery.

### *In vitro* cytotoxicity studies

**Figure 6:**
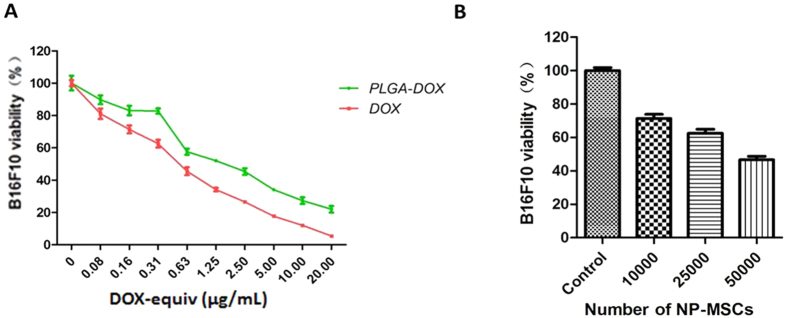
*In vitro* cytotoxicity of NP-MSCs. (**A**) Dose-response curves for cytotoxicity of DOX solution and PLGA-DOX nanoparticles with B16F10. (**B**) Cytotoxic potential of NP-MSCs was determined by MTT assay with B16F10 seeded in the receiver chamber. Data were presented as mean ± standard deviation, (n = 6 replicates for each group).

To determine the *in vitro* cytotoxic potential of NP-MSCs with B16F10 cells, different numbers of NP-MSCs were added on the top chamber of a Transwell plate, with B16F10 cells in the bottom chamber. Then, MTT analysis of B16F10 viability was conducted after 24 h treatment. Figure 6B illustrated the dose-dependent inhibition in cell survival of NP-MSCs. IC_50_ of DOX and DOX in nanoparticles was 0.47 ± 0.10 μg/mL, 1.01 ± 0.12 μg/mL, respectively, in B16F10 cells (Fig. 6A).

### The penetration of MSCs into tumor spheroid

**Figure 7:**
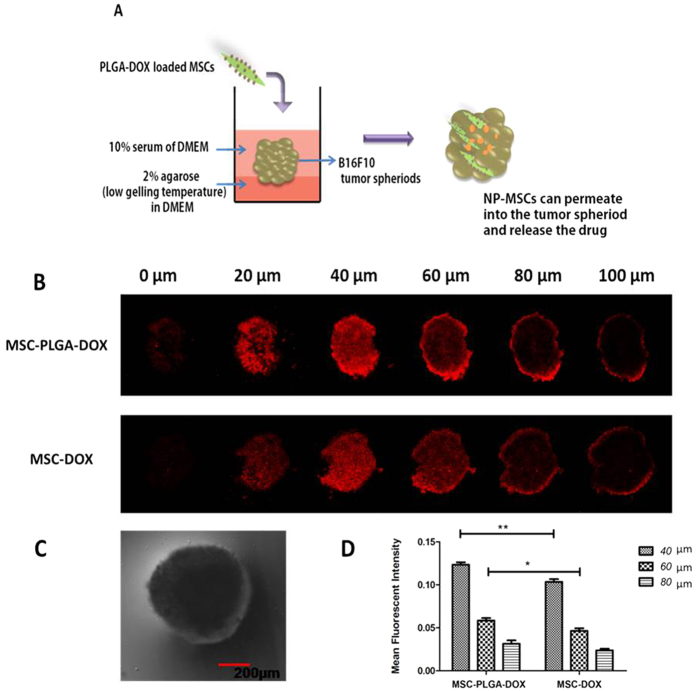
Penetration of loaded MSCs into B16F10 tumor spheroids. (**A**) Schematic diagram of experiment design. (**B**) Confocal laser scanning images of B16F10 tumor spheroids after incubated with DOX or PLGA-DOX loaded MSCs for 48 h ranging from 0–100 μm. (**C**) A representative image of B16F10 tumor spheroid in bright field with a spherical shape. (**D**) Mean fluorescence intensity calculated by Image J software at the depth of 40, 60, 80 μm. Data were presented as mean ± standard deviation, (n = 3 replicates for each group). *p < 0.05, **p < 0.01.

B16F10 tumor spheroids were established to evaluate the penetration capability of DOX-MSCs or NP-MSCs. The penetration was scanned by CLSM at different depth ranging from 0–100 μm and the mean fluorescence intensity at 40, 60, 80 μm were analyzed with Image J software. As seen in Fig. 7B, red fluorescence of DOX-MSCs or NP-MSCs appeared inside tumor mass, demonstrating their substantial penetration into tumor nest. The attenuation in mean fluorescence intensity was recorded when the depth was increased in both groups. In comparison with MSC-DOX group, the mean fluorescence intensity of PLGA-DOX loaded MSCs was relatively higher in each scanned depth, especially at 40 μm (*p* < 0.01) and 60 μm (*p* < 0.05).

### *In vivo* distribution of nanoparticles loaded MSCs

**Figure 8:**
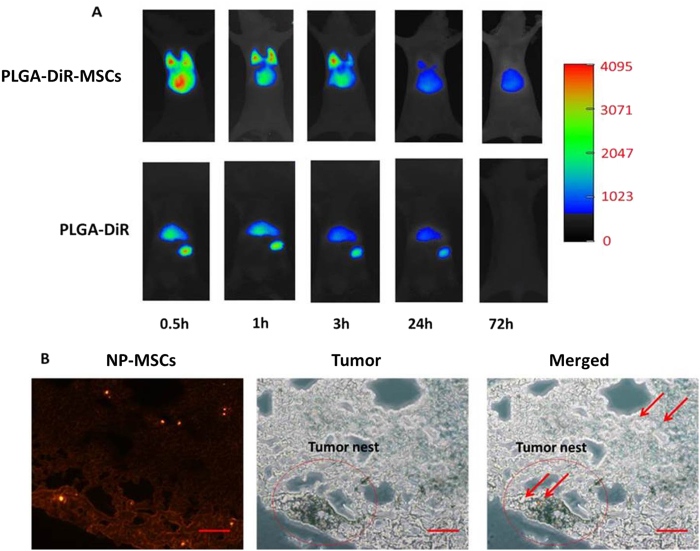
*In vivo* distribution of NP-MSCs. (**A**) *In vivo* fluorescence images of MSCs-PLGA-DiR and PLGA-DiR in lung melanoma metastasis-bearing mice after intravenous injection at determined time points (0.5, 1, 3, 24, 72 hrs). (**B**) Fluorescence images of frozen lung section with PLGA-DOX loaded MSCs on day 3 after drug injection. (Scale bar = 100 μm).

For *in vivo* investigation, DiR was used as the near—infrared fluorescent probe. The distribution of PLGA-DiR-MSCs and single nanoparticles were tracked in lung melanoma metastasis-bearing C57BL6 mice as shown in Fig. 8A. As the fluorescent images clearly displayed, PLGA-DiR-MSCs remained in the lung for at least 3 hrs after systemic injection implemented and fluorescence was still detectable after 24 hrs. On the contrary, bare nanoparticles could only be tracked in liver and spleen. Furthermore, in a separate experiment, frozen lung sections prepared on day 3 after NP-MSCs injection further demonstrated the tumor homing and penetrative feature of MSCs. DOX fluorescent was found to be distributed in tumor tissues (Fig. 8B).

### *In vivo* anti-tumor efficacy of loaded MSCs

**Figure 9:**
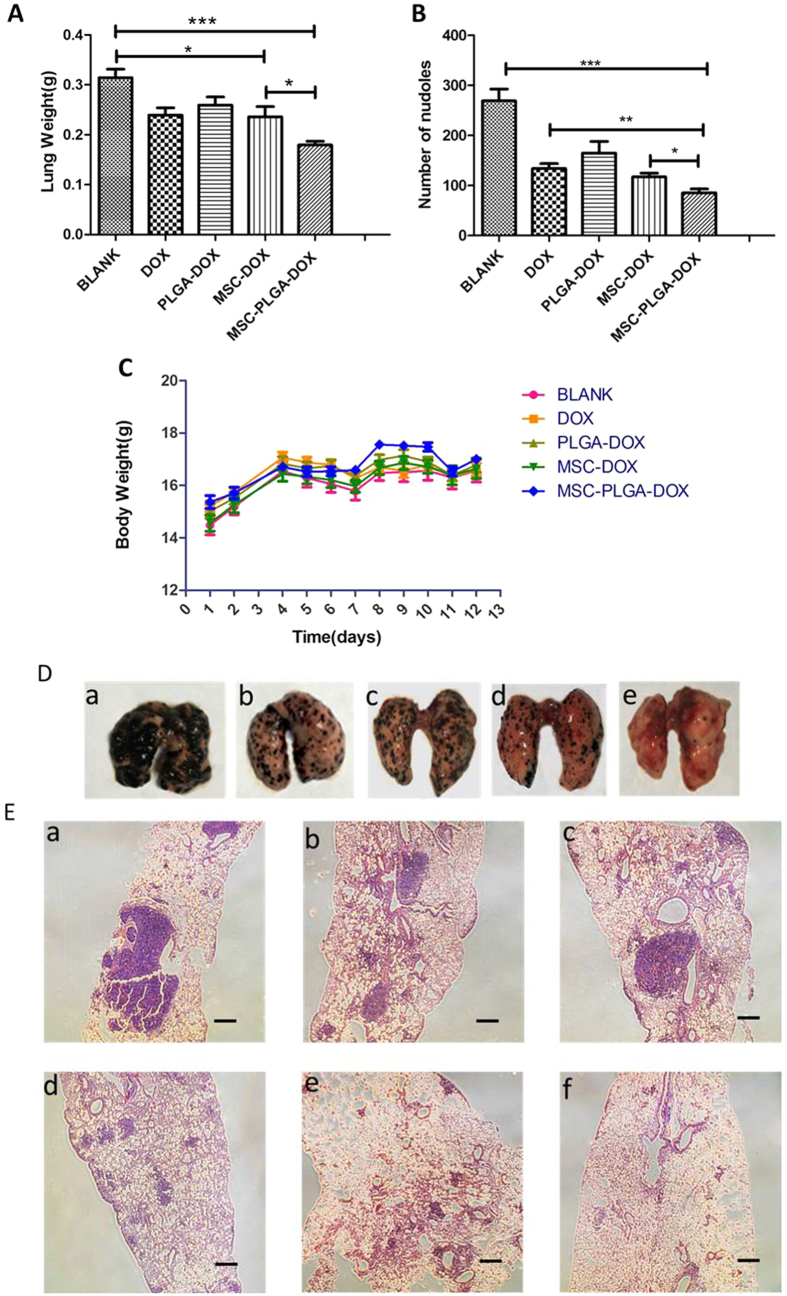
*In vivo* anti-tumor effects. (**A**) Lung weight of each group. Lungs with more metastatic colonies are usually heavier than lungs with fewer colonies. (**B**) Number of metastatic colonies of each group after treatment. (**C**) The change of body weight after B16F10 implantation. (**D**) Photographs of lungs in each treatment group. (**E**) Representative lung sections with H&E staining after treatment with (a) Blank, (b) DOX, (c) PLGA-DOX, (d) MSCs-DOX, (e) MSCs-PLGA-DOX, (f) Normal. (Scale bar = 200 μm).Data were presented as mean ± standard deviation, (n = 6 animals for each group). *p < 0.05, **p < 0.01, ***p < 0.001.

The anti-tumor effect of loaded MSCs was evaluated on C57BL6 mice with an aggressive murine melanoma pulmonary metastasis. Body weights were monitored during the experiment as an indication of safety. As Fig. 9C indicated, the body weights showed a slight fluctuations during the treatment period but were not significantly different from one another.

Shown on Fig. 9D, on day 13 of tumor inoculation, metastasized colonies spread all over the surface of the excised lungs in mice treated with saline, implying the strong pulmonary metastasis potential of B16F10 melanoma cells. The suppression of tumor metastasis induced by DOX was found to be more robust than PLGA-DOX, which was consistent with the *in vitro* results. Because of active migration and penetration potential of MSCs, NP-MSCs could improve the drug concentration in lungs and sites of the metastasis, thereby enhancing anti-tumor efficacy sequentially. The data in Fig. 9B demonstrated that mice treated with NP-MSCs had a significant reduction of lung metastases (*p* < 0.001). Also, the pulmonary weight in Fig. 9D further supported the antitumor efficacy of NP-MSCs.

Lungs in each group were also investigated by H&E staining. As seen in Fig. 9E, many melanomas appeared in the lung sections of melanoma-bearing mice, especially in the saline group. In contrast, the lungs of mice treated with NP-MSCs showed considerable reduction in melanoma number, and many normal pores also existed.

## Discussion

Virtually all, most nanomedicine including those decorated with specific binding ligands could accumulate passively in tumor tissues via EPR effect^20^. However, the accumulation of nanomedicine under ERP effect was gradually recognized to be restricted to tumors that are highly vascularized. As a result, under-perfused or hypoxic locations within tumors rarely benefit from EPR effect^21^. MSCs have shown potent tumor-homing potential in response to pro-inflammatory cytokines in tumor microenvironment, which enable the possibility of true, active carriers for tumor-targeting therapy^22^.

As it reported, MSCs have been engineered for delivery of chemotherapeutic drugs such as paclitaxel, gemcitabine and doxorubicin. Commonly-used materials in the nanoparticles engineered MSCs include polymeric micelles^23^, mesoporous silica^24^, dendrimers^25^. However, most nano-loaded MSCs were administrated intratumorally due to the limited drug loading or targeting efficiency, so that this system is not suitable for orthotropic tumor or metastases models. Some efforts have been made in lung melanoma metastases by incorporating PTX with MSCs directly. However, the anti-tumor effect was still hindered by low drug loading efficiency of 2.5 ± 0.05 pg/cell^26^, which is potentially attributed to the efflux outside the cells by the P-glycoprotein overexpressed on MSCs. MSCs modified genetically were also investigated in pulmonary melanoma metastases model. As it documented, bone marrow-derived MSCs were transfected with adenovirus vector to express CX3CL1 and inhibit the development of B16F10 lung metastases, the number of metastatic nodules treated with MSCs-CX3CL1 was significantly reduced by 84% compared with PBS group^27^. To avoid safety problems caused by virus transfection, non-viral vectors were designed for MSCs gene-modification and endowing MSCs the potent anti-tumor efficacy in syngeneic lung melanoma metastases model^28^,^29^,^30^,^31^. Nevertheless, most current researches on these non-viral vectors are still restricted to the *in vitro* evaluation of their transfection efficiency^32^. Here, we reported for the first time, the application of mice adipose-derived MSCs were loaded with doxorubicin-containing PLGA nanoparticles (PLGA-DOX) for the treatment of pulmonary B16F10 melanoma metastases. The average DOX content was measured 20.98 ± 4.02 pg/cell in NP-MSCs extremely higher than that reported in the previous work. Due to the active migration and penetration potential of NP-MSCs, drug concentration was improved in the lungs and sites of the metastasis, which resulted in enhanced anti-tumor efficacy.

In our research, controlled-release function of PLGA nanoparticles and the migratory potential of MSCs were utilized to construct a carrier-in-carrier delivery system. In the preparation of PLGA-DOX, emulsion evaporation method was employed with sodium carboxymethyl cellulose (SCMC) as the stabilizer. SCMC could adsorb on the surface of PLGA to provide ample carboxylic acid, which facilitates the loading of amide-containing drugs, resulting in high encapsulation efficiency.

MSCs uptake of DOX solution and PLGA-DOX nanoparticles are governed by passive diffusion and endocytosis, respectively^33^. Different from passive diffusion, latter manner was energy consuming, as a result, drug loaded in MSCs could get higher in DOX group when the concentration was increased, while PLGA-DOX group reached the equilibrium between endocytosis and exocytosis.

On the other side, because of the limiting number and density of MSCs in intravenous injection, enough nanoparticles incorporation in MSCs is essential for achieving high concentration in tumor tissues. Initial study showed that loading efficiency of PLGA-DOX nanoparticles reached 90.52 ± 0.63% and the drug loaded in each cell was 20.98 ± 4.02 pg/cell approximately, comparable to DOX group under the optimal incubation condition. With the addition of lung targeting, therapeutic dose could be obtained. Further conduction of nanoparticles optimization allowing more MSCs take-up and reducing the number of NP-MSCs during treatment period still needs to be investigated to improve the clinical safety.

Furthermore, the sensitivity of MSCs to DOX and PLGA-DOX were evaluated. Due to P-glycoprotein drug efflux transporter overexpression, MSCs were notably resistant to some chemotherapeutic agents^26^. Our result showed that DOX and PLGA-DOX were both non-toxic to MSCs at 25 μg/mL. At 50 μg/mL of chemotherapeutic agents, MSCs viability was revealed higher than 90% for PLGA-DOX group after 24 h drug incubation, suggesting the majority of MSCs remained viable following loading and hold the inherent traits^24^.

The crucial property of MSCs as drug carriers is their migratory and permeability potential^34^. As documented, MSCs are drawn to TNF-α, TGF-β, and other cytokines secreted in tumor microenvironment and then home to tumor tissue^35^. In this project, B16F10 cancer cells were used as chemoattractant and the tropism of MSCs was demonstrated in comparison with 0.5% FBS control. While the migratory capacities of NP-MSCs were slightly affected but still retain the innate tumor homing nature. This discrepancy can be attributed to nanoparticles attached on the membrane of MSCs or inside MSCs hampering their deformation to migrate through transwell membrane. *In vivo* distribution suggests that NP-MSCs could localize in lungs and more than 95.99% drug was released here according to the release profile.

Additionally, the permeability of loaded MSCs was investigated with an *in vitro* 3D multicellular tumor spheroid model, which mimicked physiological tumor tissue microenvironments by organized extracellular matrix and acidic pH. In the spheroid depth of 40 and 60 μm, the mean fluorescence intensity of NP-MSCs group was measured relatively stronger than DOX-MSCs, potentially due to the powerful penetration ability of NP-MSCs. Early release of DOX in medium prior to MSCs permeation into tumor spheroid may also contribute to the result. Our studies demonstrated that PLGA-DOX loaded MSCs not only retain their tumor homing nature, but also could permeate into tumor aggregates.

*In vivo* anti-tumor efficacy of NP-MSCs was carried out in C57BL6 mice bearing B16F10 melanoma that metastasized to the lungs. NP-MSCs exhibited potent anti-tumor efficacy, with the main conclusions as follows. Drug loaded MSCs retained the tumor tropic and permeability trait, which facilitated an accurate targeting of metastases and permeation of tumor nest. Moreover, DOX loaded in MSCs was mainly bumped out of cells by P-gp system and then easily eliminated from tumor mass, while nanoparticles were found to be retained in MSCs for extended periods and act as drug depots which could release drug slowly.

## Conclusions

In our experiment, MSCs isolated from adipose tissue in C57BL6 mice were loaded with PLGA-DOX nanoparticles to treat pulmonary metastases. The key properties of MSCs such as their tumor homing and permeable properties were found unaffected by drug loading, which facilitated an accurate targeting of metastases and permeation of tumor nest. Further, *in vivo* distribution studies showed that, NP-MSCs were noted to preferably reside in lungs while bare nanoparticles deposited mainly in liver and spleen. According to the photographs of lung sections and tumor spheroid scanning, PLGA-DOX carried by MSCs could penetrate into tumor nest. MSCs carrying PLGA-DOX nanoparticles were found to be effective in killing B16F10 melanoma cells *in vitro* with a dose dependent manner. *In vivo* antitumor investigations further supported the efficacy of NP-MSCs in the treatment of pulmonary metastases. Thus in this novel work, we demonstrate PLGA-DOX loaded MSCs can be promising agent for the treatment of pulmonary tumors through intravenous injection.

## Materials and Methods

### Materials

Doxorubicin hydrochloride (DOX-HCl) was purchased from Beijing Huafeng United Technology. PLGA (Mw: 15000; Carboxyl-terminated 50: 50; inherent viscosity: 0.18 dl/g) was obtained from Shandong Institute of Medical Equipment. Near-infrared dye DiR was purchased from Beijing Fanbo Biochemicals.

### Cell culture

Mesenchymal stem cells derived from the adipose tissue of C57BL6 mice were cultured in DMEM medium (Gibco, USA) containing 15% fetal bovine serum (FBS) (Gibco, USA), penicillin (100 IU/mL), streptomycin (100 μg/mL). The mouse melanoma cell line (B16F10) was obtained from ATCC (Manassas, USA) and cultured in DMEM medium containing 10% FBS and antibiotics. All cells were cultured at 37 °C in a humidified incubator containing 5% CO_2_/95% air.

### Preparation of drug-loaded PLGA nanoparticles

Doxorubicin and DiR were loaded in PLGA nanoparticles by an emulsion evaporation method^36^,^37^. In brief, 4 mg of DOX-HCL was first converted into free base with 0.01 mol/L NaOH solution and then added directly into 2 mL of acetone containing 30 mg PLGA. Next, 2.5 mL mixture of PLGA and the drug was added slowly into 0.01% sodium carboxymethyl cellulose. Followed by the evaporation of the acetone with gentle stirring overnight, the resulting DOX-PLGA nanoparticles were washed by ultrafiltration technique (500 μL, 30k MWCO, Millipore, USA) at 3000 rpm for 25 minutes to remove unloaded doxorubicin. After the final wash, nanoparticles in the upper chamber of the ultrafiltration centrifuge filter were harvested. Unloaded doxorubicin was in the lower chamber. Nanoparticles containing DiR was also prepared similarly.

### Characterization of nanoparticles

The morphology of nanoparticles was acquired using transmission electron microscope (TEM, Hitachi H-7650 and JEOL JEM-2100F, Japan) and the sample was diluted 5 times by loading sample solution on nickel grids without staining.

Particle size (diameter, nm), polydispersity index (PDI) and zeta potential (surface charge) were determined by dynamic light scattering (DLS) technique (Zeta Plus Brookehaven Instrument, UK). The PLGA NPs were diluted by deionized water before measurement, and then the analysis was performed at 25 °C with a scattering angle of 90°.

Encapsulation efficiency (EE) and drug loading coefficient (DL) were determined by ultrafiltration technique and calculated by equation (1) and (2), respectively.


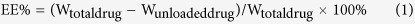



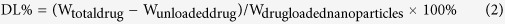


W_total drug_ and W_unloaded drug_ were determined by measuring the absorbance at 480 nm with spectrophotometric method (Microplate reader, Synergy 2, BioTek^®^ Instruments Inc, USA).

### *In vitro* release of doxorubicin from PLGA-DOX nanoparticles

The release kinetics of PLGA-DOX *in vitro* was conducted using dialysis method^38^, and PLGA-DOX resuspended in citrate buffer (3 mL, pH 5.0, 6.0 and 7.0, respectively) was transferred into dialysis bags (MWCO: 14 kDa) which was placed in 40 mL of the same buffer. The sample was then incubated at 37 °C, 100 rpm. Sink conditions were maintained throughout the experiment. At predetermined time points, 1 mL dialysate was collected and analyzed using spectrophotometric method at 480 nm. The dialysate was then replenished with the same volume of citrate buffer.

### Cytotoxicity of drug on MSCs

The cytotoxicity of DOX, PLGA, PLGA-DOX on MSCs was measured with MTT assay. Briefly, the MSCs were seeded at a density of 8 × 10^3^ cells/well on 96-well plates and incubated at 37 °C, 5% CO_2_ for 24 h. When cells reached 70–80% confluence, the medium was replaced with DOX (0.78–200.00 μg/mL), PLGA-DOX (0.78–200.00 μg/mL, DOX-equiv) and PLGA (0.01–1000.00 μg/mL), respectively. After 24 h incubation, 200 μL MTT (0.5 mg/mL) in fresh serum-free medium was added into each well. After 2–4 h, the medium was replaced by 150 μL DMSO and the colorimetric measurements were performed at 570 nm by microplate reader.

### Uptake of nanoparticles to MSCs

To investigate the optimal incubation time, 1 × 10^5^ MSCs were incubated in PBS with 20 μg/mL (DOX-equiv) DOX and PLGA-DOX. Flow cytometric analysis (FACSCalibur, USA) was performed at 15 min, 30 min, 1 h, 2 h, 3 h, 4 h after drug loaded MSCs were washed with ice cold PBS 2 times. Drug loaded MSCs were detected in the FL-2 channel depending on the red fluorescence of DOX. In another experiment, the effect of drug concentration was analyzed. DOX and PLGA-DOX with the concentration ranged from 10 μg/mL to 100 μg/mL were incubated with 1 × 10^5^ MSCs for 1 h, followed by flow cytometric test after samples were washed.

### Quantitative determination of drug loading content in MSCs

NP-MSCs were prepared by co-incubation of 5 × 10^5^ MSCs and 50 μg/mL (DOX-equiv) DOX or PLGA-DOX for 1 h. Total drug content in loaded MSCs was measured by high-performance liquid chromatography (HPLC) (LC-2010C, Japan) with the concentration range from 0.25 to 10.00 μg/mL. After the cells were washed and counted, 200 μL of cells lysis buffer was added to obtain the cell lysate. The lysate was subsequently extracted with 800 μL methanol by vortex mixing for 5 min. After centrifugation for 5 min with 12000 rpm, the supernatant was analyzed for DOX content determination with HPLC separation using fluorescence detection (λ_ex_ = 480 nm, λ_em_ = 560 nm) (RF-20A, Japan). The mobile phases were 0.01 mol/L KH_2_PO_4_: methanol: aceticacid = 40: 60: 0.4 (v/v/v) with C18 column (Agilent).

### Drug release from drug loaded MSCs

PLGA-DOX or DOX loaded MSCs were washed 3 times and then transferred into PBS (1 × 10^5^ cells in 1 mL). After culturing for 30 min, 1 h, 4 h, 6 h, 24 h, 48 h at 37 °C, the cell sample was centrifuged at 300 × g for 5 min and 600 μL supernatant was collected for HPLC analysis. The percent of drug release from NP-MSCs at each time point was calculated and plotted.

### *In vitro* migration of NP-MSCs

The migratory potential of unloaded or loaded MSCs were determined with 24-well transwellplate (PET membrane, 8 μm pore size, Corning). Following wash with PBS and resuspension in serum-free medium, 2.5 × 10^4^ NP-MSCs were added into the top chamber of the transwell plate. Then, DMEM with 0.5% serum or 0.5% serum containing B16F10 were added into the bottom chamber. Untreated MSCs served as control. After 24 h incubation, cells that did not migrate through the pores were removed with cotton swabs. Then, cells on the lower surface of the membrane were fixed in methanol for 30 min and stained with 0.1% crystal violet for 20 min. Finally, for quantification, OD value at 570 nm was measured with microplate reader after cells were destained with glacial acetic acid.

### *In vitro* cytotoxicity assessment

The anti-tumor effect of NP-MSCs on B16F10 was conducted by using a Transwell assay. B16F10 cells (2 × 10^4^) were seeded at the bottom chamber of the Transwell plates. Then, NP-MSCs of different concentration (0, 1 × 10^4^, 2.5 × 10^4^, 5 × 10^4^) were added into the top chamber of the Transwell plates. After 24 h incubation, the viability of B16F10 was determined by MTT assay.

In addition, the cytotoxicity of DOX solution and DOX loaded nanoparticles to B16F10 was also conducted. The cell viability data was analyzed using GraphPad Prism software to calculate the IC_50_ of different groups.

### Melanoma tumor spheroid penetration

The *in vitro* three-dimensional spheroids model of B16F10 cells were employed to evaluate the penetrability of loaded MSCs. 2% (w/v) agarose solution were prepared in serum-free DMEM by heating at 80 °C for 20 min and then sterilized. 5 × 10^3^ tumor cells with complete medium were seeded into a 96-well plate which was initially coated with agarose to avoid cell adhesion. The tumor spheroids were grown at 37 °C in 5% CO_2_ for 5 days until the diameter of the spheroid reached 600 μm. These compact and uniform spheroids were then used for subsequent permeation studies.

MSC-DOX and MSC-PLGA-DOX were co-cultured with tumor spheroids for 48 h. After incubation, tumor spheroids were washed twice with PBS and fixed with 4% paraformaldehyde for 20 min. The permeability of drug was then investigated with confocal laser-scanning microscopy (FV1000, Olympus, Japan).

### *In vivo* distribution studies of nanoparticles loaded MSCs

The distribution of drug loaded MSCs in mice bearing lung melanoma metastasis was investigated with DiR as a fluorescent probe. PLGA-DiR-MSCs were injected intravenously at 1 × 10^6^ cells/200 μL/mice. *In vivo* distribution of PLGA-DiR-MSCs was detected at determined time points (0.5, 1, 3, 24, 72 h) in a dark room with NIR imaging system. Tumor bearing mice injected with PLGA-DiR nanoparticles solution were imaged as a control.

Additionally, PLGA-DOX loaded MSCs (NP-MSCs) were also injected into tumor bearing mice for lung frozen specimen observation on day 3 under a fluorescence microscope (IX53, Olympus, Japan).

### *In vivo* anti-metastasis effect

All care and handling of animals were carried out according to the international laws and policies (EEC Council Directive 86/609, 1987) and approved by the animal ethics committee of China Pharmaceutical University (Nanjing, China). The lung metastases models were established in six-week-old female C57BL6 mice by intravenously inoculation of 1 × 10^6^ B16F10 cells through a tail vein (0 day). The mice were then randomly divided into five groups (n = 6 animals for each group): Blank group (Saline), DOX group (DOX), PLGA-DOX group (PLGA-DOX), MSC-DOX group (MSC-DOX), and MSC-PLGA-DOX group (MSC-PLGA-DOX). All the doxorubicin preparations were injected via the tail veins at the dose of 2 mg/kg on the 3rd, 7th, 11th day. On 13th day of tumor inoculation, lung specimens in all groups were harvested and photographed. Lung weight and the number of metastatic nodules were also examined. The lung specimens were finally treated with formalin for hematoxylin and eosin (HE) staining.

### Statistical analysis

Statistical analysis was performed using GraphPad Prism 6.0 and at least three independent experiments were carried out. Data are presented as mean ± standard deviation, and are analyzed using paired student’s t-test and one-way analysis of variance (ANOVA).The significant difference level was set at *p* < 0.05.

## Additional Information

**How to cite this article**: Zhao, Y. *et al*. Targeted delivery of doxorubicin by nano-loaded mesenchymal stem cells for lung melanoma metastases therapy. *Sci. Rep.* **7**, 44758; doi: 10.1038/srep44758 (2017).

**Publisher's note:** Springer Nature remains neutral with regard to jurisdictional claims in published maps and institutional affiliations.

## Figures and Tables

**Figure 1:**
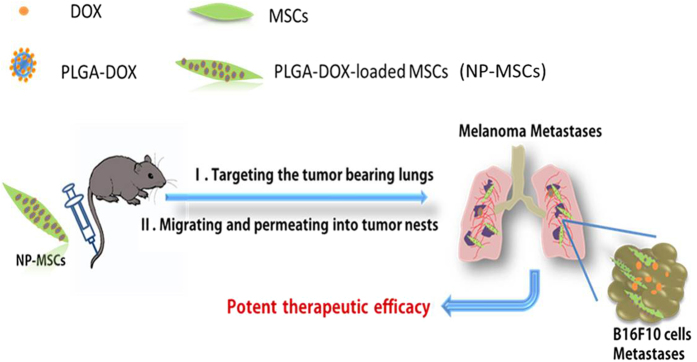
MSCs integrated with drug containing nanoparticles were applied in the treatment of pulmonary B16F10 melanoma metastases.

**Table 1:** Physicochemical characteristics of PLGA-DOX and PLGA-DiR (n = 3 replicates for each group).

Sample	Particle Size (nm)	Zeta Potential (mV)	Polydispersity index (PDI)
PLGA-DOX	150.30 ± 2.70	−37.46 ± 2.07	0.15 ± 0.04
PLGA-DiR	198.50 ± 3.30	−34.25 ± 11.42	0.15 ± 0.03
